# Auricular Acupressure Combined with Self-Help Intervention for Treating Chronic Tinnitus: A Longitudinal Observational Study

**DOI:** 10.3390/jcm10184201

**Published:** 2021-09-16

**Authors:** Winfried Schlee, Jorge Simoes, Rüdiger Pryss

**Affiliations:** 1 Department of Psychiatry and Psychotherapy, University of Regensburg, 93053 Regensburg, Germany; jorge.simoes@klinik.uni-regensburg.de; 2 Institute of Clinical Epidemiology and Biometry, University of Würzburg, 97080 Würzburg, Germany; ruediger.pryss@uni-wuerzburg.de

**Keywords:** tinnitus, acupressure, self-help, ecological momentary assessment, stress

## Abstract

Tinnitus is a phantom sound perception in the ears or head and can arise from many different medical disorders. Currently, there is no standard treatment for tinnitus that reliably reduces tinnitus. Individual patients reported that acupressure at various points around the ear can help to reduce tinnitus, which was investigated here. With this longitudinal observational study, we report a systematic evaluation of auricular acupressure on 39 tinnitus sufferers, combined with a self-help smartphone app. The participants were asked to report on tinnitus, stress, mood, neck, and jaw muscle tensions twice a day using an ecological momentary assessment study design for six weeks. On average, 123.6 questionnaires per person were provided and used for statistical analysis. The treatment responses of the participants were heterogeneous. On average, we observed significant negative trends for tinnitus loudness (Cohen’s *d* effect size: −0.861), tinnitus distress (*d* = −0.478), stress (*d* = −0.675), and tensions in the neck muscles (*d* = −0.356). Comparison with a matched control group revealed significant improvements for tinnitus loudness (*p* = 0.027) and self-reported stress level (*p* = 0.003). The positive results of the observational study motivate further research including a randomized clinical trial and long-term assessment of the clinical improvement.

## 1. Introduction

Tinnitus is a condition associated with a continuous noise in the ears or head and can arise as a symptom of many different medical disorders. The condition is very common with a prevalence of 10–15% [1], but the fundamental mechanisms of tinnitus are still incompletely understood. Although many patients are not unduly troubled, others find the disorder life-changing. These severe cases of tinnitus can be accompanied by anxiety, depression, insomnia, and concentration problems, all of which can impair quality of life.

There is little evidence for effective tinnitus treatments and no licensed pharmacological therapy has been found. The Cochrane Library currently lists nine completed systematic reviews on different tinnitus treatments; namely Tinnitus Retraining Therapy (TRT), Cognitive Behavioral Therapy (CBT), hyperbaric oxygen therapy, sound therapy (masking), hearing aids, repetitive Transcranial Magnetic Stimulation (rTMS), ginkgo biloba, anticonvulsants, and antidepressants (www.cochranelibrary.com (accessed on 16 September 2021)). In patients with severe and profound sensorineural hearing loss, it was demonstrated that cochlear implantation can lead to a long-lasting reduction of tinnitus symptoms (see e.g., [2]). However, in the vast majority of tinnitus patients, the hearing loss is not so severe that a cochlear implant is clinically advised. For this general tinnitus patient population, no uniformly effective treatment for tinnitus is identified. One of the important reasons for the lack of clinical evidence is the large heterogeneity of tinnitus cases. Elgoyhen et al. [3] highlighted the large patient heterogeneity to be one of the major reasons for inconsistent results in studies on tinnitus. Tinnitus can be described on various dimensions such as its etiology, perceptual characteristics of the sound (i.e., pitch and loudness), time since onset, continuous or intermittent sound perception, levels of conscious awareness and perceived distress, and comorbidities. A review by Baguley and colleagues [4] identified at least 13 different types of causal factors that can be responsible for tinnitus. Hearing loss is the most commonly known risk factor and can result from acute noise trauma, occupational or leisure noise exposure or age-related hearing loss. Somatic tinnitus has been suggested as another cause for tinnitus, where altered muscle tension in the neck or jaw can contribute to the development of tinnitus [5].

In addition, there is a complex bidirectional interaction between tinnitus and stress. Tinnitus patients often perceive their tinnitus as stressful, intrusive and annoying and a considerable subgroup develops insomnia, attentional and psychological problems such as anxiety or depression as a consequence of the ongoing tinnitus perception [6,7]. A bidirectional interaction is suggested because stress can also aggravate tinnitus perception. Many patients report that the onset of their tinnitus was preceded by stressful events [8,9,10]. The stress level and the affective state mediate the relationship between the loudness of the tinnitus and the individually perceived distress of tinnitus [11]. Furthermore, dynamics of emotions are associated with the course of tinnitus over time [12], which adds to the complex interaction of tinnitus distress, tinnitus loudness, stress, and emotional perception. This complex interaction was also observed during the first wave of the COVID-19 pandemic. Beukes et al. 2020 [13] and Schlee et al. 2020 [14] both investigated the influence of the pandemic on the individual suffering by the chronic tinnitus patients and reported highly hetereogeneous results.

The challenge in tinnitus research is to address this patient heterogeneity by carefully considering the inter-subject differences of the chronic tinnitus patients and draw generalizable logical conclusions from it. It is, therefore, important to analyse the idiosyncratic influence of different factors on the individually perceived tinnitus, apply statistical analysis on the single subject level—and derive nomothetic models at the group level that can be generalized to a larger patient group.

With this analysis, we want to propose a methodological approach that applies statistical analysis at a single case level to consider the patient’s heterogeneity and synthesizes the results at a group level. During a period of six weeks, the patients applied an intervention where it was suspected that the impact on the tinnitus will be heterogeneous. Information about the tinnitus, stress, emotional states, neck and jaw muscle tensions were collected on a daily basis with high ecological validity in the daily environment of the tinnitus patients. The trajectories of tinnitus and related factors were analyzed at a single subject level, categorized into different patient groups, and all results described at the group level. A prerequisite for statistical analysis at the subject level is a large number of data samples over a longer period of time. We used an Ecological Momentary Assessment (EMA) research design where the participants are asked to fill out a short questionnaire one or more times a day. The questionnaire is delivered by a smartphone app to the smartphone owned by the participants and notifications where send as reminders on a regular basis.

The new tinnitus treatment combines an auricular acupressure treatment with self-help intervention. While the auricular acupressure treatment for tinnitus is a new approach, self-help treatment in tinnitus has a long tradition with self-help groups organized by national tinnitus patient associations for several centuries. A systematic review by Nyenhuis et al. [15] reported that self-help interventions reduced tinnitus distress and depression. However, an understanding of the intervention mechanism is still lacking. Greenwell and colleagues [16] emphasize that more high-quality research on self-help with better reporting will be needed. In recent years, self-help interventions for tinnitus are implemented in various e-health applications. An overview is given by Kalle et al. 2018 [17].

Based on pilot data, we assumed the treatment response to be heterogeneous with some participants benefiting from a robust reduction of tinnitus symptoms while other participants benefit only to a small extent or not at all. The aim of this study was to investigate the change of tinnitus and related symptoms during a six week treatment period applying statistical analysis on a single subject level and group level.

## 2. Materials and Methods

### 2.1. Study Description

We are reporting a longitudinal observational study investigating the temporal development of tinnitus and related symptoms during a combinational treatment of six weeks. The combined treatment consists of an acupressure device behind and around the ear, marketed as ForgTin, together with a smartphone app that provides self-help tips to the participants. Using an Ecological Momentary Assessment app, the participants provided self-reports about tinnitus loudness, tinnitus stress, mood, stress level, tensions in the jaw and neck muscles as outlined in Table 1. In addition to these questions, participants answered if they were using the acupressure device and entered comments in a free text field, which are not analyzed here. The study was approved by the ethical review board of the University of Regensburg (protocol number: 15-101-0204). Participants did not have a financial benefit when participating in the data collection.

The treatment and app assessment were done under naturalistic conditions and the data collection was reduced to a minimum to assess the development of tinnitus and related symptoms with high ecological validity and low intrusion to everyday life. Only the short questions within the app had to be answered by the participants. On a voluntary basis, the participants were asked to fill out the Tinnitus Sample Case History Questionnaire (TSCHQ, [18]) before the start of the treatment phase. 

### 2.2. Combined Treatment

**Figure 1:**
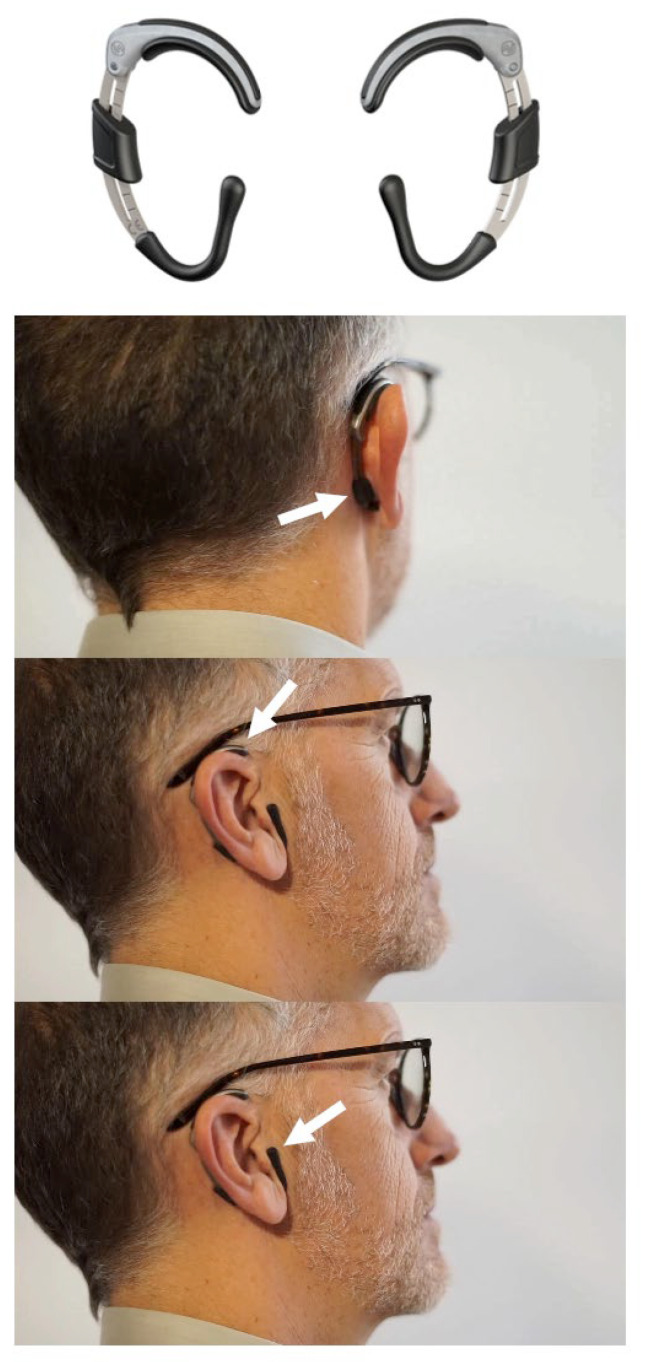
Acupressure device. Picture of the acupressure device (top) and how it should be mounted around the ear. The white arrows point to the parts of the device that can be adjusted to the individual ear.

During the six week treatment period, the participants used an acupressure device which applies a soft pressure at various points around the ear. The acupressure device, marketed as ForgTin, can be used on one or both ears and can be adapted to the individual shape and size of the ear. The device can be adapted to the individual shape of the ear. At the upper end of the device there is a bracket that can be moved up and down to adjust to the individual ear size. The thickness of the bracket is about 6 mm at the anterior end and 11 mm at the posterior end. Behind the ear, between helix and skull a thin, a curved and stiff metal piece of 1 mm thickness extends toward the ear lobe. A silicone plug with the thickness of 5 mm is attached to the metal piece in the region of the posterior auricular muscle. At the lower part of the device, the stiff metal piece tapers into a flexible and silicon-coated metal that wraps around the ear lobe forward to the tragus. The bracket, the silicone plug and the flexible metal that extends to the tragus can be adjusted to the individual ear. Participants were asked to adjust the device to avoid strong pressure and pain. Pictures of the device and how it is mounted around the ear are shown in Figure 1. To the best of our knowledge, there is no scientific study testing auricular acupressure in tinnitus. As inclusion criteria for the analysis, only those participants were included that used the acupressure device at least 50% of the days during the six weeks period. Device usage was reported via the smartphone app. Within the same app, the participants also received self-help tips for better coping with tinnitus. The tips were randomized from a library of 50 tips. The tips included e.g., suggestions for improving the sleep, reducing stress, improve concentration, relaxing despite the tinnitus sound, or improving the general health. Every week, the participants received a new tip in the app.

### 2.3. Statistical Analysis

Statistical analyses were conducted in R [19] using R-version 4.0.3 (10 October 2020). Linear regression models were calculated for each participant to predict the symptom (e.g., tinnitus loudness) based on the duration of the intervention, measured in days. Average regression estimates are reported, and the distribution of the slopes visualized. Non-parametric tests (Wilcoxon rank-sum test) were calculated to test the null hypothesis that the distributions of the slope estimates are identical in the study group and the matched control group. Cohen’s *d* effect size was calculated using the effsize package [20]. Missing values were coded as NA and removed for the respective analysis. Only participants with at least six weeks of EMA sampling were included. A time window of six weeks was used for all participants to standardize the length of the observation period.

## 3. Results

### 3.1. Characteristics of Study Participants

Ecological momentary assessment data were collected from 39 tinnitus participants, which used the acupressure device and the smartphone app for at least six weeks. During the six weeks period, the participants provided on average 123.6 EMA questionnaires per person reporting about their tinnitus, related symptoms, and device usage. The participants purchased the acupressure device in an ordinary online store and agreed that their data can be used for scientific purposes. As listed in Table 2, the participants used the acupressure device on 86.9% of the reported days. In the remaining 13.1%, the participants used the app, but not the acupressure device. The characteristics of the study participants are reported in Table 2. Participants were free to provide information about their age, sex, and duration of tinnitus. Overall, 30 out of 39 participants provided their age, 22 out of 39 gave information about their sex, and 29 of 39 answered the question about their tinnitus duration. EMA data were collected from all participants and was analyzed anonymously.

### 3.2. Description of the Individual Tinnitus Trajectories and Trend Analysis

The individual data of participants have been visualized and are provided in the Supplemental Material S1 for visual inspection. As expected, the development of tinnitus and related symptoms is highly heterogeneous with some patients showing a strong decrease of tinnitus symptoms while others remain relatively stable over the duration of six weeks. For each participant, the linear trend of the tinnitus measures and related symptoms were analyzed separately. A linear regression was calculated for each participant to predict the symptom (e.g., tinnitus loudness, tinnitus distress, etc.) based on the day of the ongoing intervention. The slope estimates are summarized in Table 3 and the intercepts are reported in the Supplemental Material S2. The trajectories of tinnitus loudness and stress showed the strongest negative slope, revealing that the respective symptom decreased over time. An average slope of −0.023 for tinnitus loudness means in this context that the loudness of the tinnitus decreased on average by 0.023 points per unit of time within the six weeks period.

The distribution of the slope estimates was tested against the null hypothesis that the median is equal to zero. This null hypothesis was rejected for the distribution of tinnitus loudness, tinnitus distress, stress, and tensions in the neck muscles. Furthermore, we calculated the Cohen’s *d* effect size, also testing against zero. The reduction of tinnitus loudness revealed a large effect size of −0.861, the reduction of stress a medium to large effect size with −0.675. We found only small effect sizes for the reduction of tinnitus distress, jaw tensions, and neck tensions (−0.478, −0.286, −0.356 respectively). The effect size for the change of mood was negligible at 0.010.

**Figure 2:**
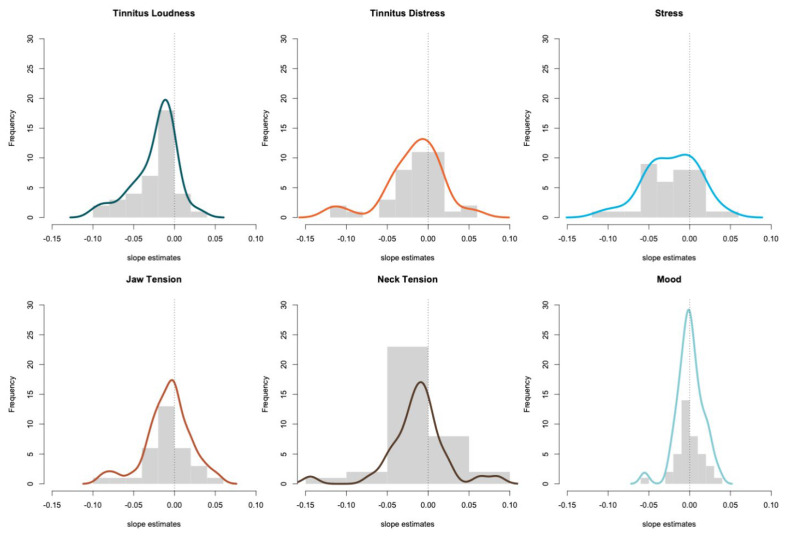
Visualization of the slope estimates in the linear regression models. A histogram of the slope estimates is shown for tinnitus loudness, tinnitus distress, stress, jaw tension, neck tension and mood. Density plots overlay the histograms.

The slope estimates for each individual participant are visualized in Figure 2. The number of participants with a significant positive or negative slope is provided in Table 4. A significant negative trend was found in 15 of the 39 participants (38.5%). A significant negative trend for tinnitus distress was found in 28.2% of the participants, in 38.9% of the participants for stress, in 40.6% of participants the jaw tensions decreased, in 38.9% the neck tensions and in 10.3% the mood increased. An overview of the statistical significance of all slope estimates is given in Table 4. Please note that a negative trend in tinnitus loudness, tinnitus distress, stress, and tensions in the jaw or neck indicates a reduction of the symptoms while for mood a positive trend can be interpreted as an improvement. In three participants, there was not enough data provided to fit a regression model. In six participants it was not possible to fit a model for the tensions in the jaw and three participants did not provide enough data for estimating a model for the neck tensions.

### 3.3. Comparison between the Study Group to a Matched Control Group

As described in Section 3.2, a negative statistical trend was found for 38.5% of the participants with respect to tinnitus loudness and for 28.2% of the participants with respect to tinnitus distress. However, it remains an open question whether these trends are different to the natural fluctuations of the symptom without intervention? No placebo-control was available due to the observational nature of the study presented in Section 3.1 and Section 3.2. Therefore, we compared the study results to matched control group from the TrackYourTinnitus study (see e.g., [21,22]). In the TrackYourTinnitus study, participants also used an Ecological Momentary Assessment smartphone app and also answered repeated questionnaires on tinnitus loudness, tinnitus distress and the perceived stress level with similar phrasing of the questions. Thirty-nine participants of the TrackYourTinnitus study were selected and matched to study group with respect to age, gender, tinnitus duration and tinnitus distress at the baseline of the EMA sampling. The participant characteristics of both groups are reported in Table 5. Causes for tinnitus and pathologies in the study group and the matched control group were diverse. In the study group, two participants reported that their tinnitus was related to a loud blast of sound, one related it to whiplash, two participants related it to a change in hearing, 14 participants mentioned stress as a cause of tinnitus, two participants reported that a head trauma was related to tinnitus, one reported bruxism as the leading cause for tinnitus, and for the remaining participants the cause of tinnitus was unknown. In the matched control group, four participants related their tinnitus to a loud blast of sound, three participants reported a change of hearing together with the onset of tinnitus, 21 participants related their tinnitus onset to stress, one participant to a head trauma, and for the remaining participants the cause of tinnitus was unknown.

**Figure 3:**
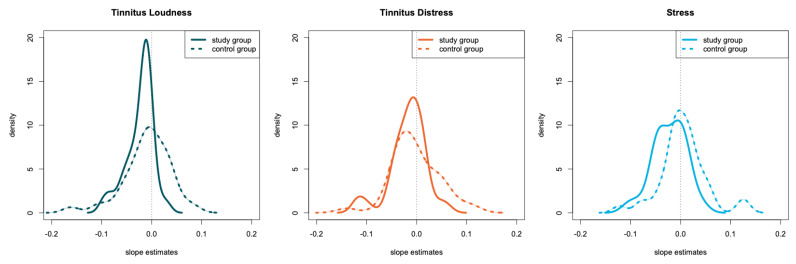
Visualization of the linear estimates of the study group and the matched control group. Estimates are shown for tinnitus loudness, tinnitus distress, and stress. Only the estimates of the statistically significant regressions are shown.

Linear regression models were fitted for the participants in the control group to predict the symptom based on the day of the ongoing intervention. We applied the same methodology to the study group. The estimated model parameters are summarized in Table 6 for both groups. Two non-parametric tests were applied to compare the distribution of model estimates between the two groups. The Wilcoxon rank-sum test is sensitive to difference in the median of two distributions testing the null hypothesis of equal distribution of the model estimates. This hypothesis was rejected when comparing the slope estimates for tinnitus loudness and stress. The trend for tinnitus loudness and stress was significantly lower for the study group, meaning that the symptom ratings decreased over time. Table 6 summarizes the regression estimates for both groups and the statistical comparison between them. The slope estimates for the individual participant of both groups are visualized in Figure 3.

## 4. Discussion

Here we presented a longitudinal observational study on participants with chronic tinnitus that used a combined treatment of acupressure around the ear plus advice for self-help interventions. Over a period of six weeks, the participants reported about their tinnitus and related symptoms via a smartphone app, which allowed tracking of these symptoms with high temporal resolution. Over the period of six weeks, the participants provided on average 123.6 self-report questionnaires. As expected, there was a high within-subject variability of the tinnitus perception as well as a high between-subject variability with heterogenous responses to the tinnitus treatment. For the statistical analysis, we took advantage of a large amount of sampling points per subject, which allowed the fitting of regression models on a single subject model.

### 4.1. Change of Tinnitus Loudness and Distress 

We observed a significant negative trend of tinnitus loudness in 15 out of 39 (38.5%) participants. A significant increase in loudness was detected in only one participant (2.6%). The data of the study group were compared to a matched control group to answer the question if such a change of tinnitus loudness can also occur under naturalistic conditions without intervention. The matched control group was selected from the TrackYourTinnitus study where the participants also used a smartphone app with an identical EMA design. Non-parametrical tests revealed a difference from the slopes for tinnitus loudness and stress between the groups.

The results for tinnitus distress; however, are weaker with only 11 participants (28.2%) showing a significant negative trend and four participants (10.3%) reporting a significant increase. The slopes of tinnitus distress were not significantly different from the slopes of the TrackYourTinnitus sample (all *p* > 0.05).

In summary, the effects of the combined treatment are stronger for the tinnitus loudness than for tinnitus distress, which is probably in favor for the tinnitus sufferers since a recent analysis by Rademaker et al. 2021 [23] concluded that tinnitus patients consider loudness the most important outcome measure. With this study, we can only document the changes of tinnitus loudness and distress but cannot identify the underlying therapeutic mechanism. The acupressure device is designed to apply soft pressure at various pre- and post-auricular points. The material of the device is flexible and can be adjusted to the individual ear. This also means that the stimulated points around the ear can slightly differ from day to day depending on the handling of the user. To the best of our knowledge, there is currently no scientific research on acupressure around the ear for treating tinnitus. In principle, it is possible that the device stimulates muscles, fascia, or lymph nodes in this area. Especially in participants with a somatic tinnitus component, a treatment that affects the jaw and neck muscles, directly or indirectly, could be beneficial. The anterior auricular muscles, the superior auricular muscle, and the posterior auricular muscle are the most likely candidates that can be influenced by the acupressure device in use here. In addition, six intrinsic muscles exist. Liguan et al. 2018 [24] outlined the neuronal connections of these muscles to the brainstem, limbic structures, and the cortex. The temporal fascia and the masseteric fascia are the most likely candidates to be influenced by the acupressure device. The posterior auricular lymph nodes and the preauricular lymph nodes are located in the region that can be stimulated by acupressure device; however, it is not clear if the gentle pressure by the acupressure device can have an effect of lymph drainage to these nodes. More research is needed to investigate the possible mechanisms on auricular acupressure treatment in chronic tinnitus. Future research needs also to disentangle the effects of acupressure and self-help tips. Therefore, it will be important to collect data if the participants read and understood the self-help tips and if they integrated the tip into their daily life. We want to mention here that there are various treatment options for somatic tinnitus currently under scientific evaluation including various devices (e.g., mandibular advancing device, MAD, [25]) or physical therapy (e.g., Conservative Temporomandibular Therapy [5]). We are optimistic that combined research efforts in this field can improve the scientific understanding of the somatic tinnitus and the therapeutic mechanisms for improving this condition.

### 4.2. Changes of Tensions in the Jaw and Neck Muscles

A significant decrease of tensions in the jaw muscles was observed in 40.6% of the participants and a decrease of neck muscle tension in 38.9%. A significant increase of jaw muscle tension was detected in 18.8% and an increase in neck muscle tensions in 11.1%. The distribution of the slope estimates for the jaw tensions was not significantly different from zero while the slope estimates for the neck tensions were significantly lower than zero, with a small effect size of −0.356. Since the jaw and neck muscles have not been investigated in the TrackYourTinnitus study, it was not possible to compare these results to a matched control group. It is, therefore, not possible to decide whether the changes are a result of the intervention or merely reflect a natural fluctuation of the symptom. With visual inspection of the single subject data, it can be hypothesized that there are participants with a temporal relationship between neck muscle tensions and tinnitus symptoms in a way that small increases in muscle tension precede an increase of tinnitus. This will be subject to further research using time series analysis.

### 4.3. Changes in Mood 

With all the statistical analysis that we calculated here, there was no hint that the mood of the participants changed systematically toward either direction. There was only a small number of patients with a significant trend, three participants (7.7%) toward a more negative mood and four participants (10.3%) toward a more positive mood. The slope estimates were not significantly different from zero and the effect size negligible (0.010).

### 4.4. Changes of Stress 

A significant decrease in the stress level was observed during the six weeks period. A significant negative trend of stress was found in 38.9% participants and a positive trend in only 5.6% of the participants. The distribution of the slope estimates was significantly different from zero with a medium to strong effect size of −0.675. The slopes of stress estimates were significantly lower than the slopes of the TrackYourTinnitus sample. The magnitude of stress changes is therefore comparable to the magnitude of changes in tinnitus loudness. This was surprising to us, especially since the reduction of stress is stronger than the reduction of tinnitus distress.

One explanation could be that the reduction of tinnitus loudness leads to a reduction of the stress level of the participant. In this case, we should see a correlation between tinnitus loudness and stress, which was not the case. Post-hoc analysis showed an average correlation between tinnitus loudness and stress of 0.29 while the average correlation between tinnitus loudness and tinnitus distress was 0.71. However, it has to be mentioned here that a correlation analysis only tests for linear relationships. At the moment, we cannot rule out that there is a more complex non-linear or temporal relationship between stress and tinnitus loudness. Another explanation could be that the repeated sampling of stress questionnaires helped the participants to better reflect on their own stress level and develop strategies for effective coping. In this case, the matched control group, which also filled out the questionnaires on stress, should also show a reduction of stress. This, however, was not the case. Therefore, we hypothesize that the stress reduction is a consequence of the intervention—either by the self-help tips or the acupressure. The library of self-help tips consisted of 50 different tips and seven of these tips contained recommendations for coping with stress. It is, therefore, likely that this kind of self-help interventions helped the participants to reduce the general stress level. Additionally, the auricular acupressure is a likely candidate for reducing the stress level. Cha and colleagues [26] conducted a randomized trial with auricular acupressure over a two-week period and reported a reduction of the self-rated stress level together with a reduction of the blood cortisol level. It is, therefore, possible that the combination of self-help tips and acupressure triggered the decrease of stress in the participants. To find out which part of the intervention contributed to the improvement, it will be of high importance to conduct a randomized clinical study with a cross-over design to systematically vary the application of the two treatment components and assess the clinical improvements and the compliance of the participants with the respective intervention.

### 4.5. Limitations

One limitation of this observational study design is the lack of a randomized control group. Although a matched control group was used for comparison, a selection bias cannot be ruled out. Therefore, more research is strongly encouraged to investigate the efficacy of the treatment with study designs that allow determining the magnitude of the treatment effect and test if the changes are clinically meaningful. A randomized control trial will be needed to investigate the treatment effects under controlled conditions and reduced bias. In addition, it will be important for the scientific understanding of the intervention to investigate the clinical efficacy of the different treatment components. Based on this data, it is not possible to identify the therapeutic effect of the treatment and it is currently unknown if there is a direct therapeutic effect to the tinnitus or if indirect effects account for the tinnitus improvement. Furthermore, an analysis over a longer time period could reveal long-term changes and test if the improvement persists over time. Such a long-term study should include non-linear analysis to model the asymptotic decline of symptoms toward the zero lines. Given the large he

Heterogeneity of treatment effects among the participants, more research is needed to identify early predictors for the treatment response. A better scientific understanding of the underlying mechanisms of the auricular acupressure and self-help intervention could help to further improve the efficacy of the combinational treatment.

## 5. Conclusions

In summary, the observational study revealed strong significant improvements of tinnitus loudness and medium to strong significant improvements of the general stress level over a period of six weeks with the combined intervention of auricular acupressure and self-help intervention. The reduction of tinnitus distress and muscle tensions in the jaw and neck was statistically significant but small. Comparison with a matched control group confirmed that the improvements in tinnitus loudness and stress are significantly different from the fluctuations that are typically observed under real-life conditions. Overall, the results of this observational study encourage further investigations. A randomized clinical trial will be needed to overcome the bias of this observational study and to identify the contribution of the two treatment components.

## Figures and Tables

**Table 1:** Questions for ecological momentary assessment.

Question Number	German	English	Answer Options
1	Wie laut empfinden Sie den Tinnitus aktuell?	How loud do you currently feel the tinnitus?	visual slider on a scale from 0 (low) to 10 (high)
2	Wie belastend empfinden Sie den Tinnitus im Moment?	How stressful is the tinnitus at the moment?	visual slider on a scale from 0 (low) to 10 (high)
3	Wie ist ihre aktuelle Stimmungslage?	What is your current mood?	5 smileys from very sad (rating 1) to very happy (rating 5)
4	Wie gestresst fühlen Sie sich gerade?	How stressed do you feel right now?	visual slider on a scale from 0 (no stress) to 10 (maximum stress level)
5	Wie verspannt fühlt sich Ihr Kiefer gerade an?	How tense does your jaw feel right now?	visual slider on a scale from 0 (no at all tense) to 10 (very tense)
6	Wie verspannt fühlt sich Ihr Nacken gerade an?	How tense does your neck feel right now?	visual slider on a scale from 0 (no at all tense) to 10 (very tense)

The participants downloaded an app for the daily assessment of tinnitus and related symptoms. The original language of the app is German. A translation of the questions is given in column 3. Answer options are displayed with English translations.

**Table 2:** Characteristics of the study participants.

	Units	Study Group
Total participants	Number of Participants	39
Sex ratio	Female:Male (% of enrolled)	54.5%:45.5%
Age at Baseline	Years (mean (SD))	50.8 ± 14.6
Tinnitus Duration	Years (mean (SD))	9.7 ± 11.9
Device Usage	% of Days (mean (SD))	86.9% (±15.3)
Sampling points during the 6 weeks period	Mean ± SD (min; max)	123.6 ± 79.4 (42; 371)

**Table 3:** Results of the linear trend analyses.

Symptom	Slope	*p* Value	Cohen’s *d* Effect Size
Tinnitus Loudness	−0.023 ± 0.027	<0.001	−0.861
Tinnitus Distress	−0.018 ± 0.036	0.005	−0.478
Stress	−0.022 ± 0.033	<0.001	−0.675
Tension in the jaw muscles	−0.008 ± 0.029	0.124	−0.286
Tension in the neck muscles	−0.013 ± 0.037	0.009	−0.356
Mood	0.000 ± 0.016	0.929	0.010

For each participant and symptom, we calculated a linear regression analysis predicting the symptom based on time. The estimates for the slope are summarized. Mean and standard deviation of the estimates are provided. *p* value is based on the Wilcoxon singed rank test which was applied to test against the null hypothesis that the median of the distribution is zero.

**Table 4:** Overview of the slope estimates in the linear trend analyses.

Symptom	Participants with A Significant Negative Trend	Participants with No Significant Trend	Participants with A Significant Positive Trend
Tinnitus Loudness	15 (38.5%)	23 (59.0%)	1 (2.6%)
Tinnitus Distress	11 (28.2%)	24 (61.5%)	4 (10.3%)
Stress	14 (38.9%)	20 (55.6%)	2 (5.6%)
Tension in the jaw muscles	13 (40.6%)	13 (40.6%)	6 (18.8%)
Tension in the neck muscles	14 (38.9%)	18 (50.0%)	4 (11.1%)
Mood	3 (7.7%)	32 (82.1%)	4 (10.3%)

For each symptom we report how many participants revealed a significant negative or positive trend. Number of participants and percentages are reported.

**Table 5:** Characteristics of the study group and the matched control group.

	Units	Study Group	Matched Control Group	*p* Value
Total participants	Number of Participants	39	39	1
Sex ratio	Female:Male (% of enrolled)	54.5%:45.5%	63.2%:36.8%	0.645
Age at baseline	Years (mean (SD))	50.8 ± 14.6	51.3 ± 12.4	0.891
Tinnitus duration	Years (mean (SD))	9.7 ± 11.9	9.7 ± 10.4	0.989
Tinnitus loudness at baseline	Points (mean (SD))	6.5 ± 2.2	6.1 ± 2.7	0.407
Tinnitus distress at baseline	Points (mean (SD))	5.6 ± 2.7	5.6 ± 2.7	0.947
Hearing problem	Number of Participants	9	11	0.795
Hearing aid users	Number of Participants	3	4	1

Tinnitus loudness and distress are reported on a scale from 0 to 10. *p* values are based on the Student’s t-test for continuous variables and the Chi square test for categorical variables.

**Table 6:** Comparison of regression estimates of the study group compared to the matched control group. *p* values are based on the Wilcoxon rank-sum test, which was calculated to compare the distributions of estimates.

Symptom	Slope Study Group	Slope Matched Control Group	*p* Values
Tinnitus Loudness	−0.023 ± 0.027	−0.009 ± 0.047	0.027
Tinnitus Distress	−0.018 ± 0.036	−0.002 ± 0.048	0.252
Stress	−0.022 ± 0.033	0.005 ± 0.047	0.003

## Data Availability

The data presented in this study are available on request from the corresponding author.

## Supplementary Materials

The following are available online at https://www.mdpi.com/article/10.3390/jcm10184201/s1. S1.: Visualization of the individual data in the study group over the six-week period. S2.: Overview of the linear regression estimated in the study group.
