# Supplementary material for: Auricular Acupressure Combined with Self-Help Intervention for Treating Chronic Tinnitus: A Longitudinal Observational Study
Source: J Clin Med. 2021 Sep 16;10(18):4201. doi: 10.3390/jcm10184201 (PMC8466364; doi:10.3390/jcm10184201)
Supplement: Supplementary file 1 [file jcm-10-04201-s001.zip › jcm-1354077-supplementary.pdf]

S1. Supplemental Material. Visualization of the individual data in the study group over the six-week period. Tinnitus loudness and tinnitus distress as the main outcome variables are drawn in thick lines.

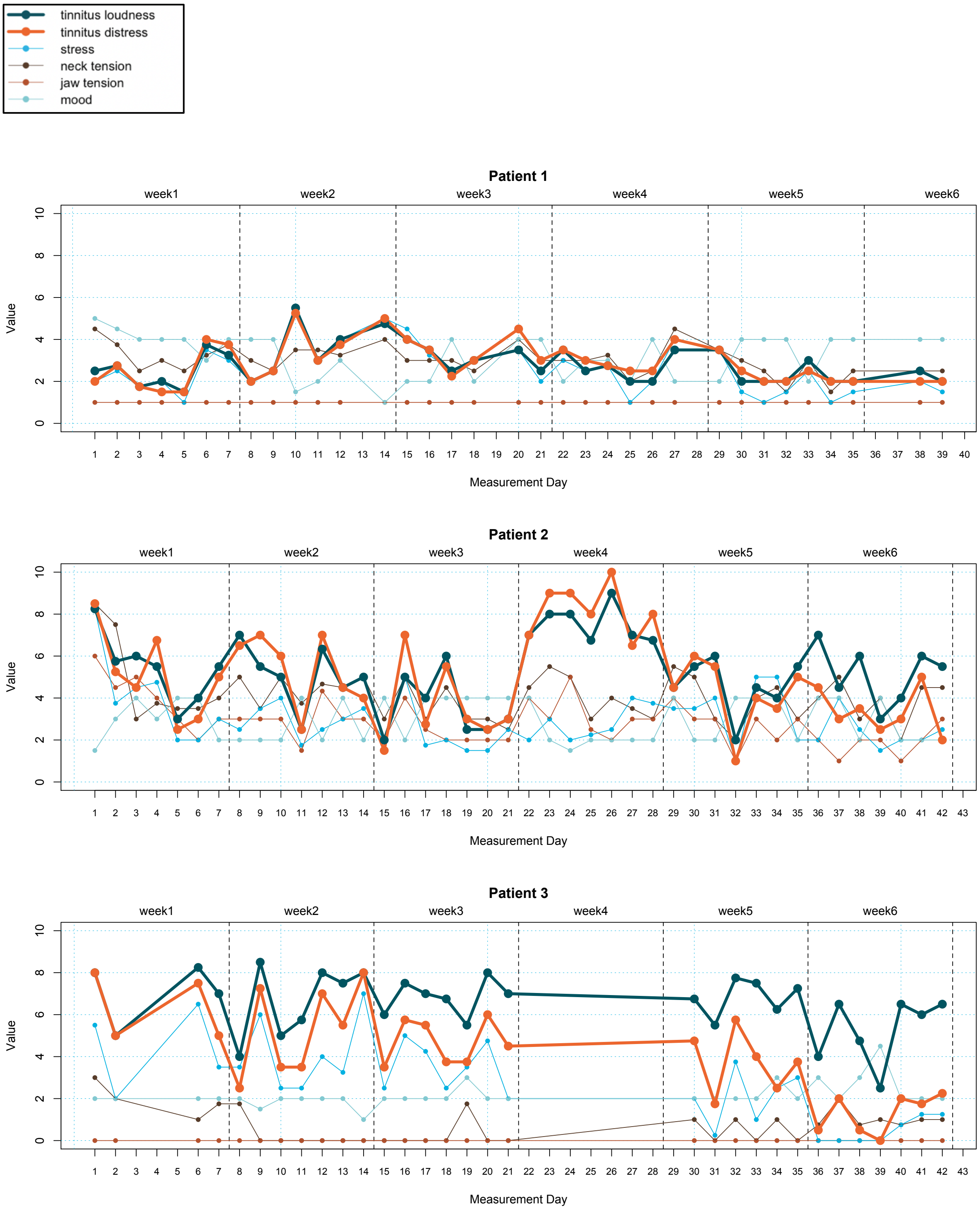

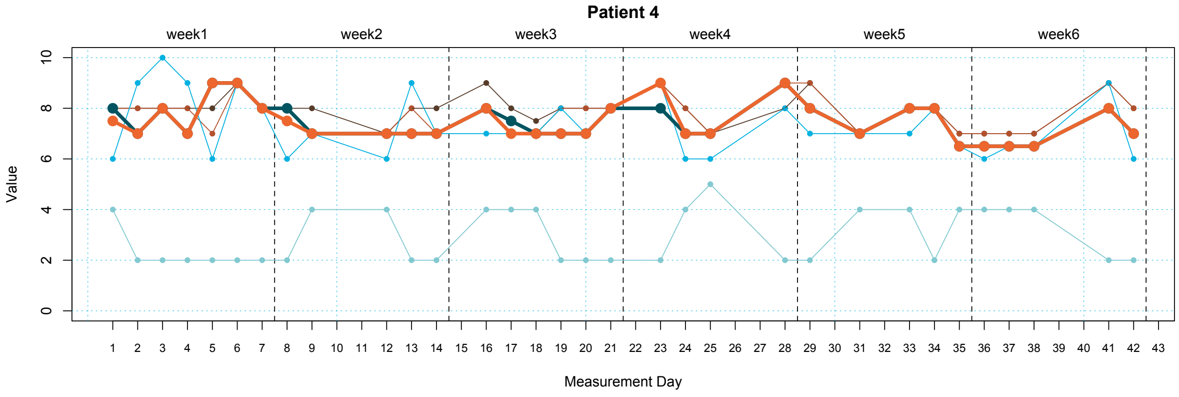
Patient 4
week1
week2
week3
week4
week5
week6
Value
Measurement Day

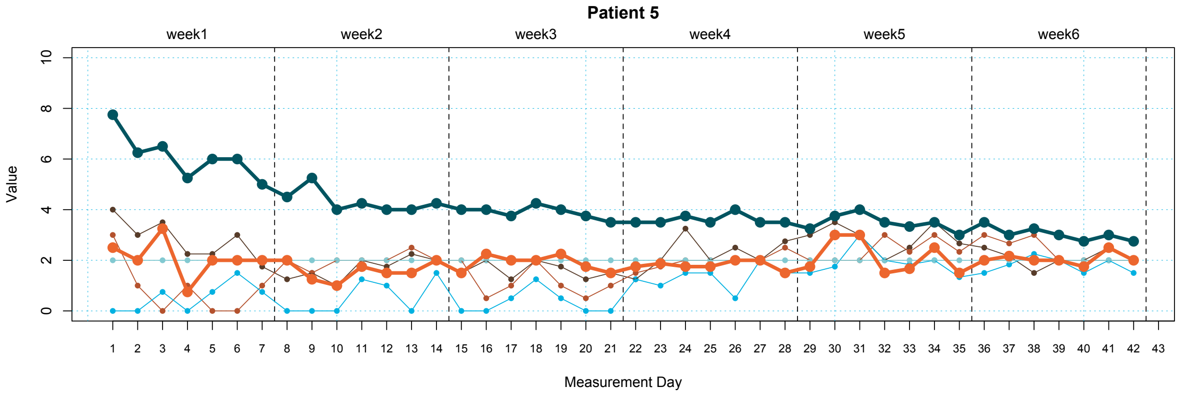
Patient 5
week1
week2
week3
week4
week5
week6
Value
Measurement Day

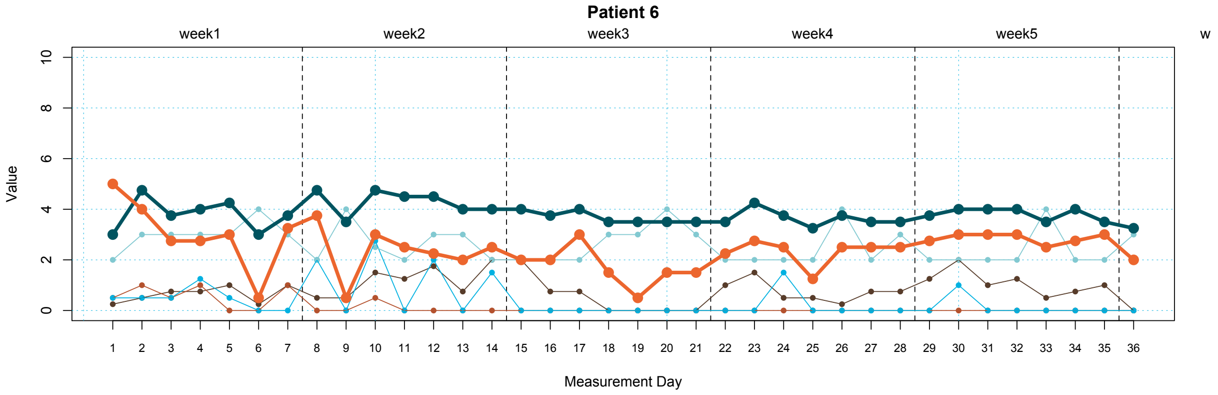
Patient 6
week1
week2
week3
week4
week5
w
Value
Measurement Day

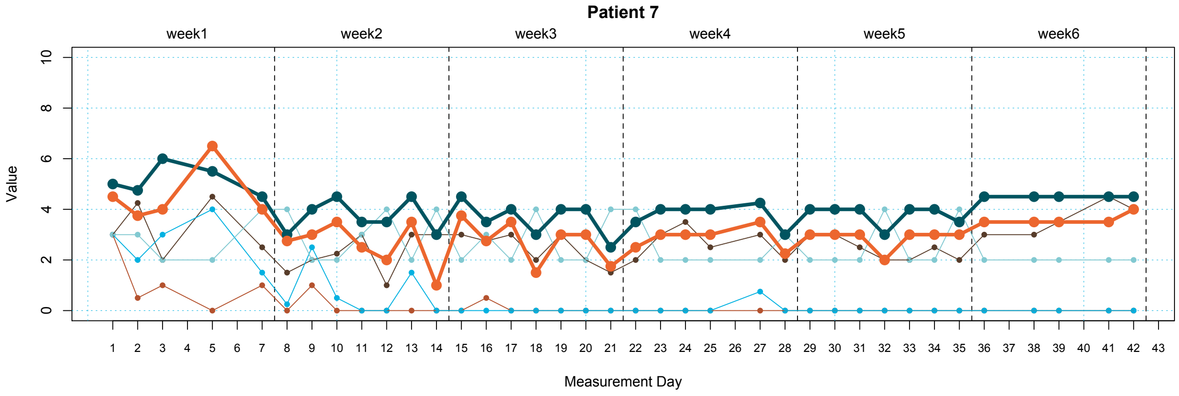
Patient 7
week1
week2
week3
week4
week5
week6
Value
Measurement Day

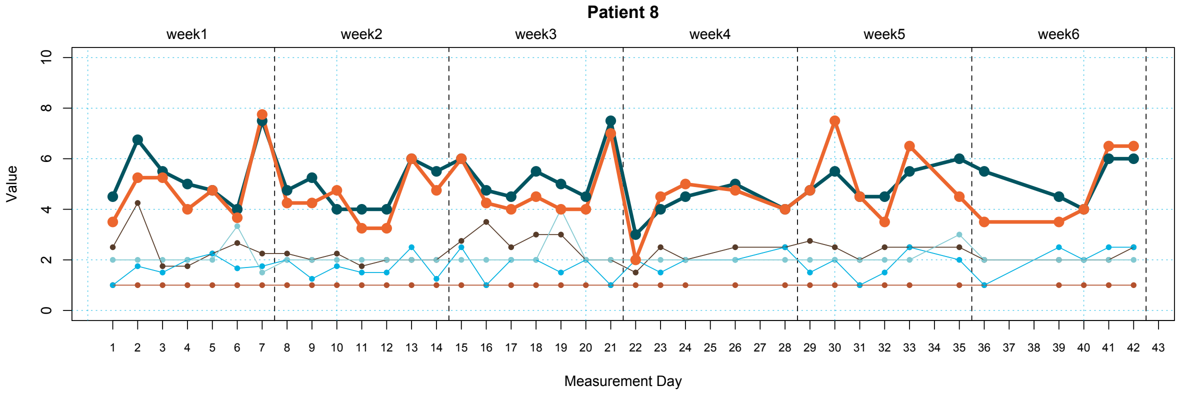
Patient 8
week1
week2
week3
week4
week5
week6
Value
Measurement Day

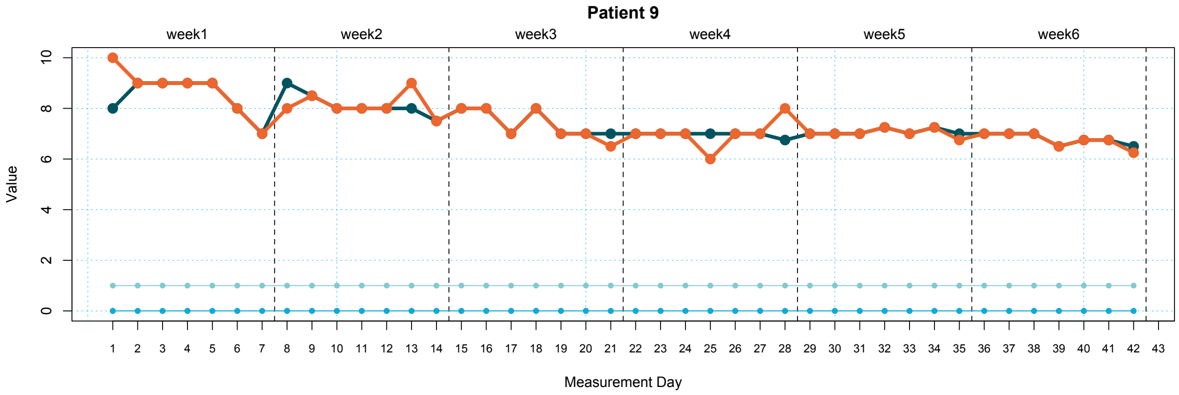
Patient 9
week1
week2
week3
week4
week5
week6
Value
Measurement Day

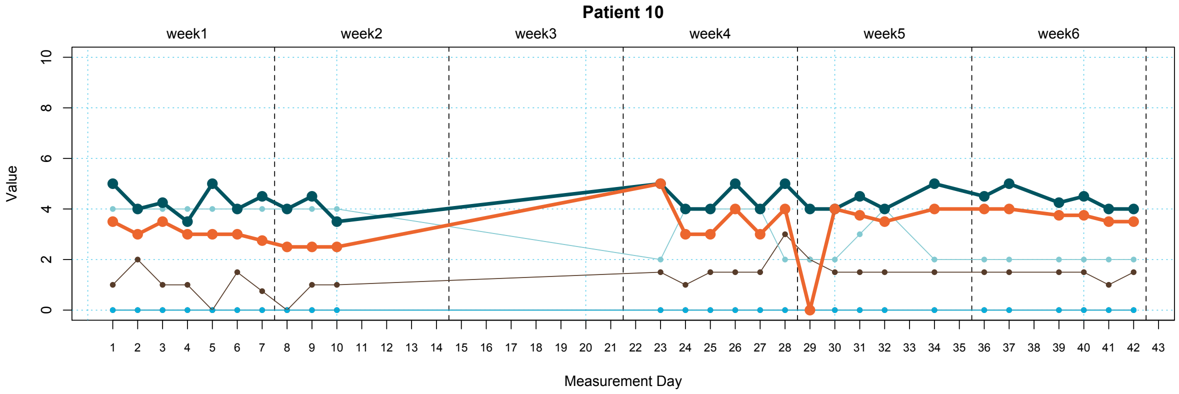
Patient 10
week1
week2
week3
week4
week5
week6
Value
Measurement Day

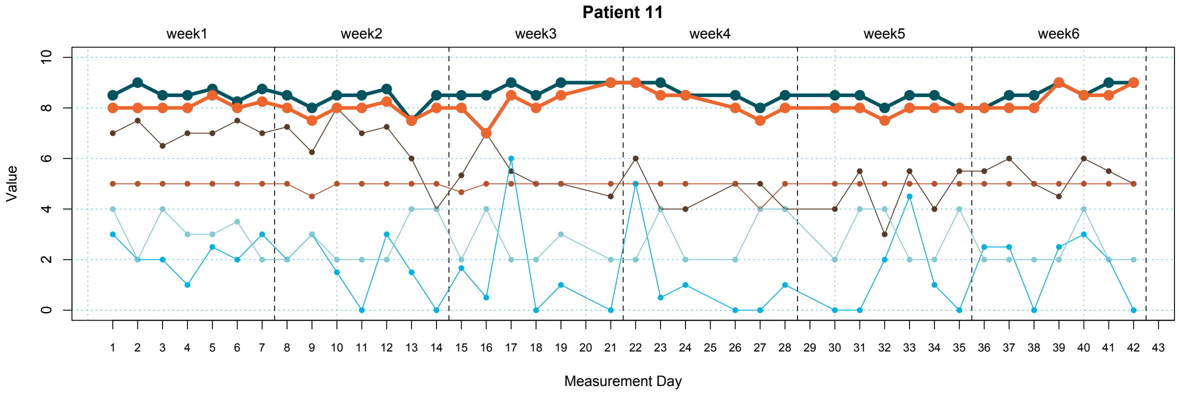
Patient 11
week1
week2
week3
week4
week5
week6
Value
Measurement Day

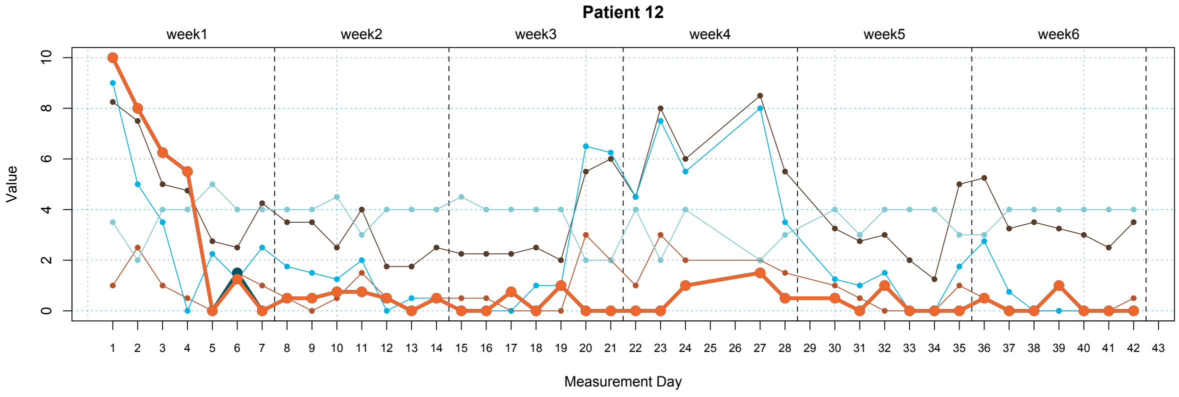
Patient 12
week1
week2
week3
week4
week5
week6
Value
Measurement Day

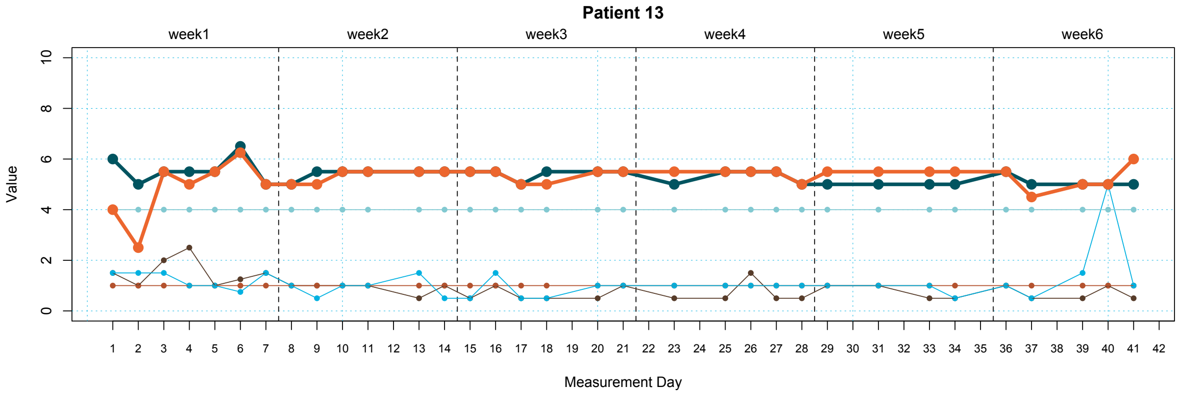
Patient 13
week1
week2
week3
week4
week5
week6
Value
Measurement Day

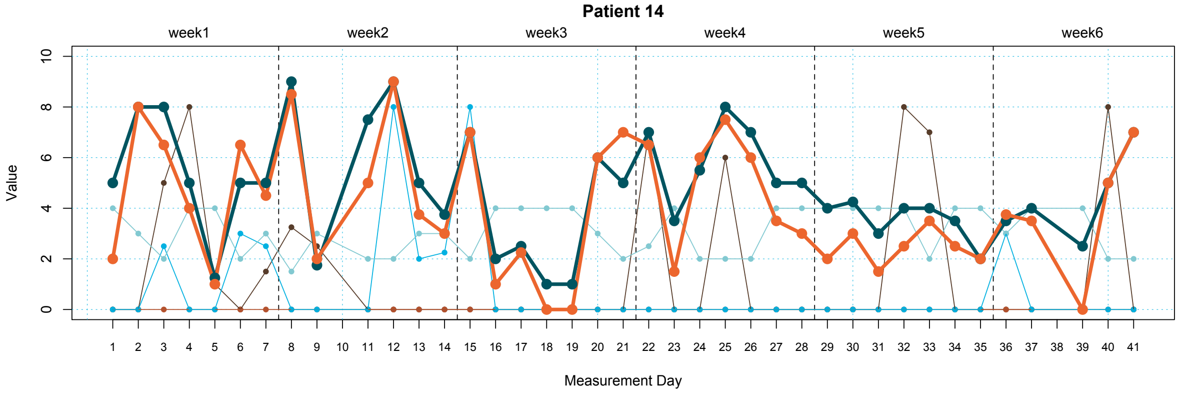
Patient 14
week1
week2
week3
week4
week5
week6
Value
Measurement Day

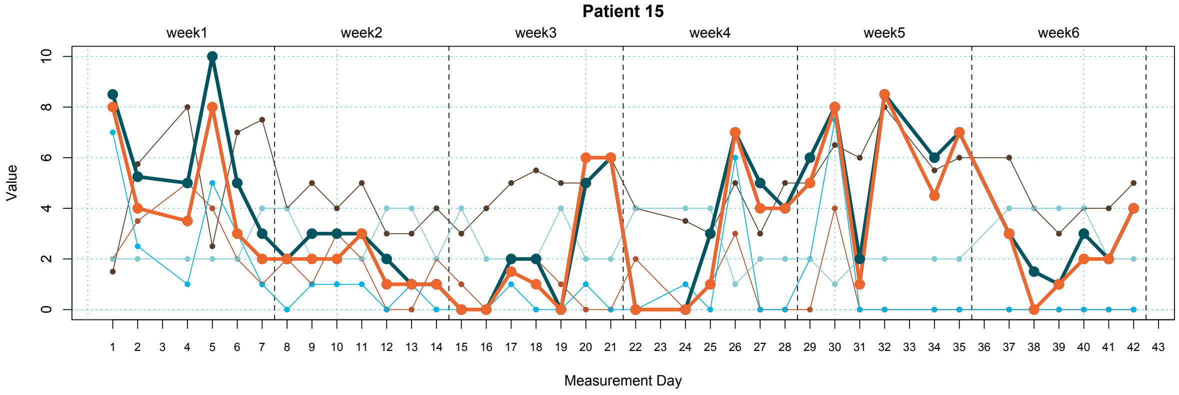
Patient 15
week1
week2
week3
week4
week5
week6
Value
Measurement Day

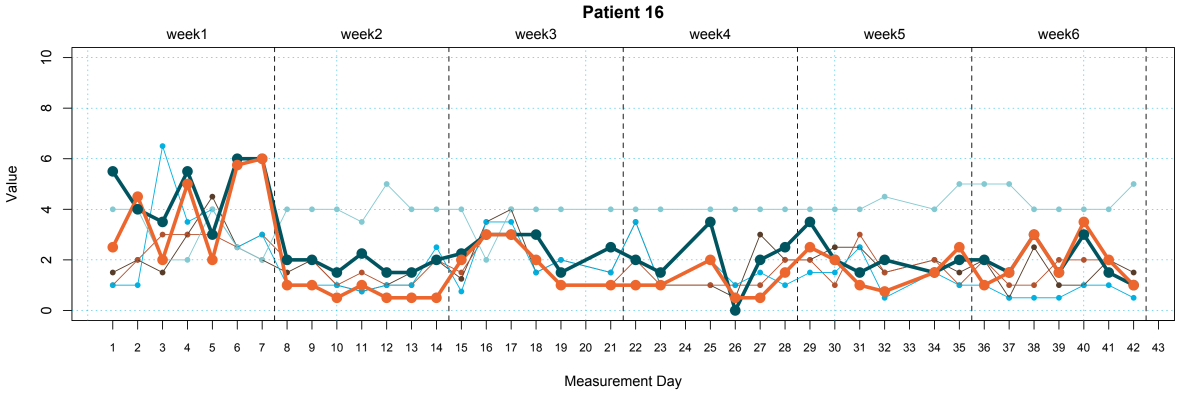
Patient 16
week1
week2
week3
week4
week5
week6
Value
Measurement Day

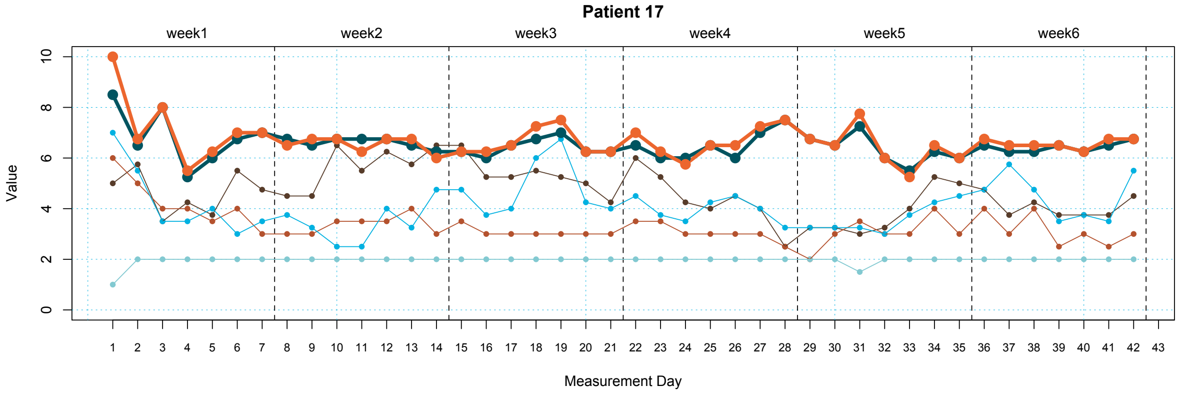
Patient 17
week1
week2
week3
week4
week5
week6
Value
Measurement Day

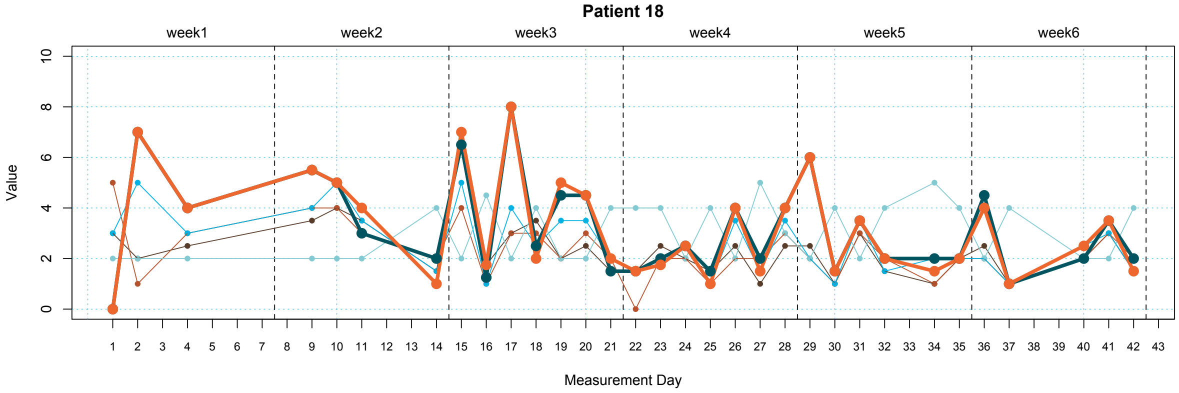
Patient 18
week1
week2
week3
week4
week5
week6
Value
Measurement Day

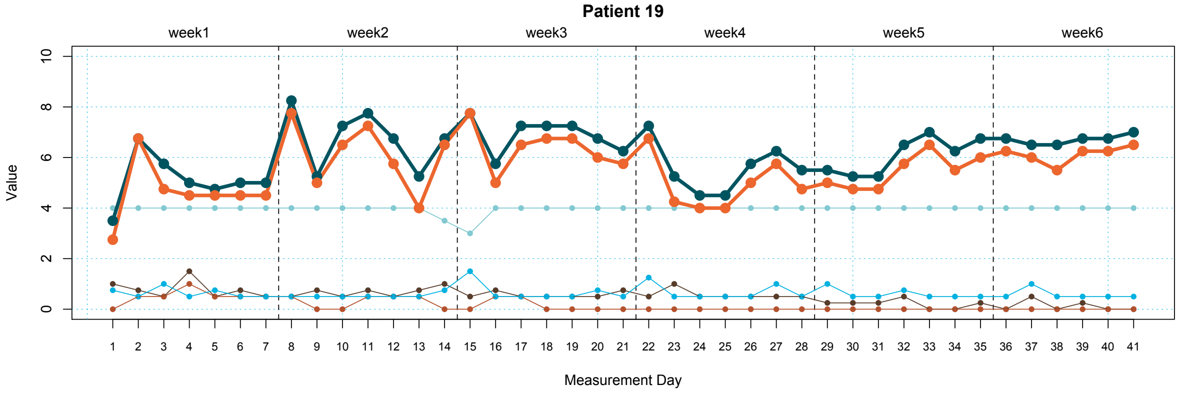
Patient 19
week1
week2
week3
week4
week5
week6
Value
Measurement Day

Patient 20

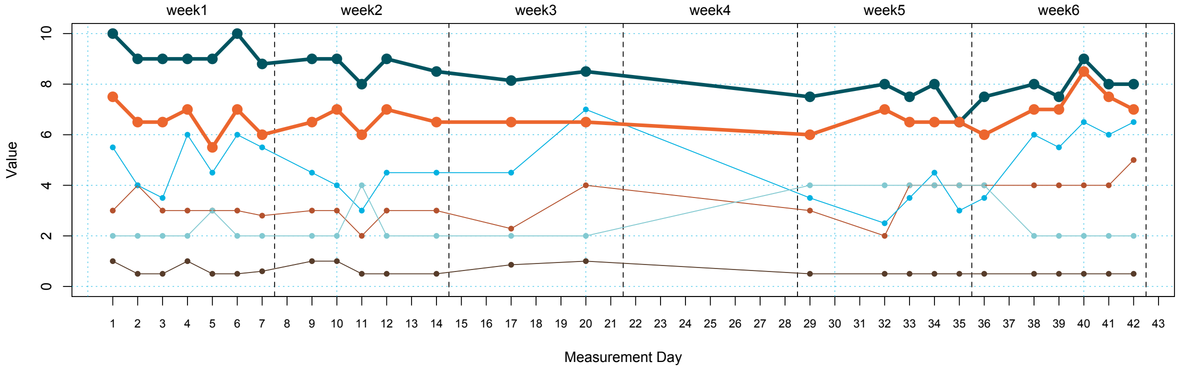

Patient 21

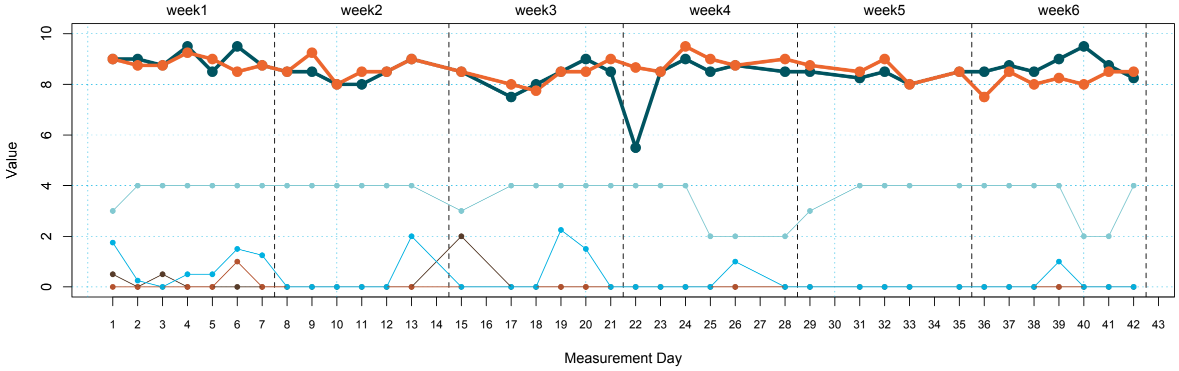

Patient 22

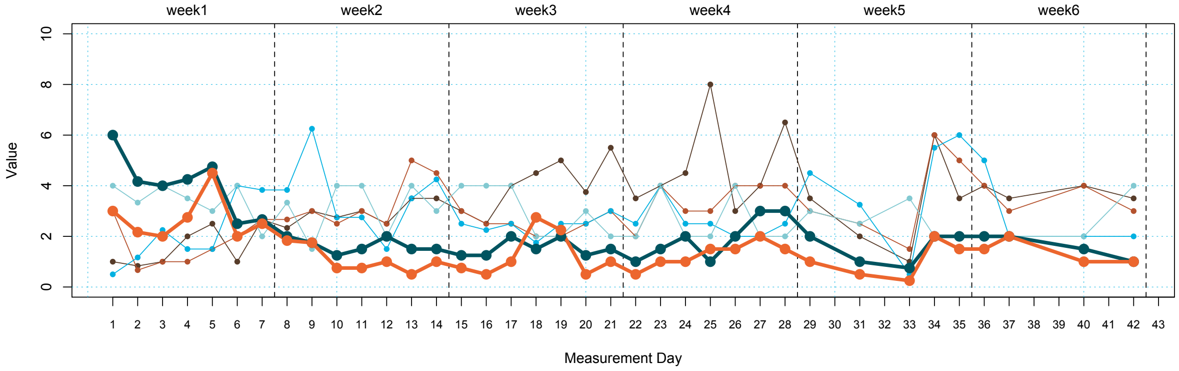

Patient 23

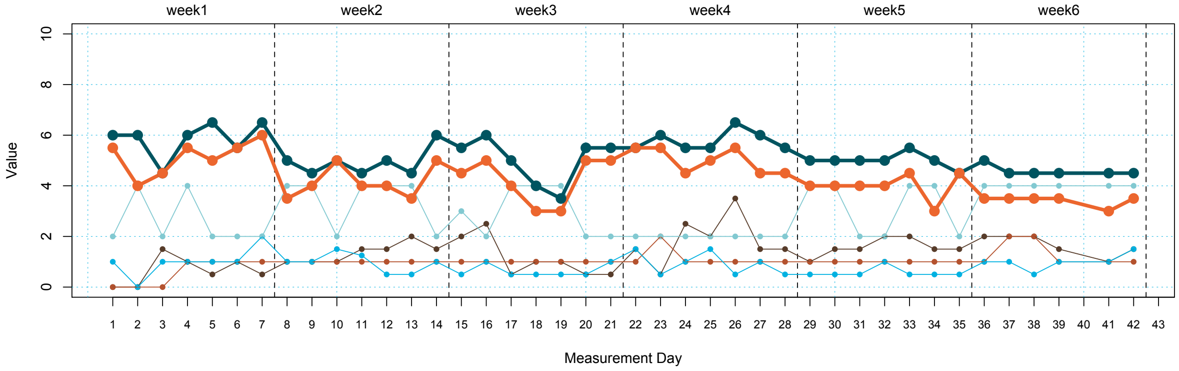

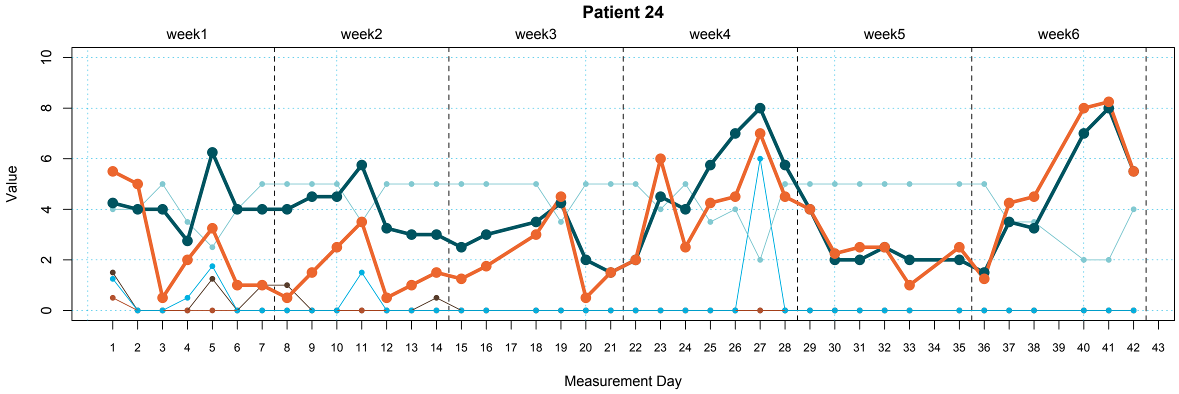
Patient 24
week1
week2
week3
week4
week5
week6
Value
Measurement Day

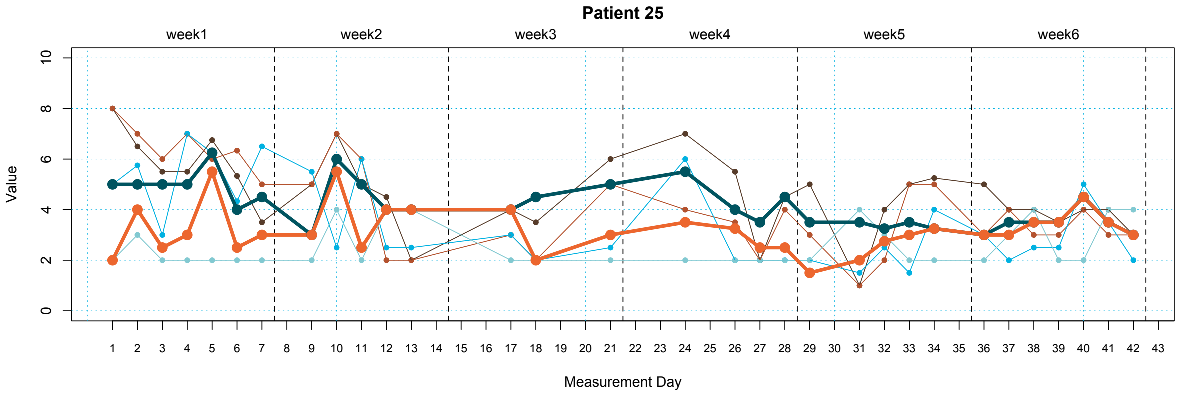
Patient 25
week1
week2
week3
week4
week5
week6
Value
Measurement Day

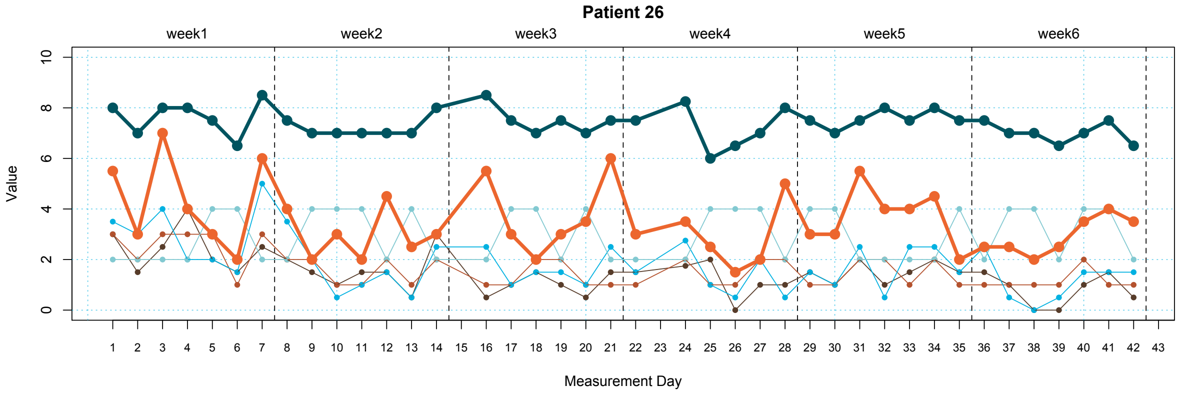
Patient 26
week1
week2
week3
week4
week5
week6
Value
Measurement Day

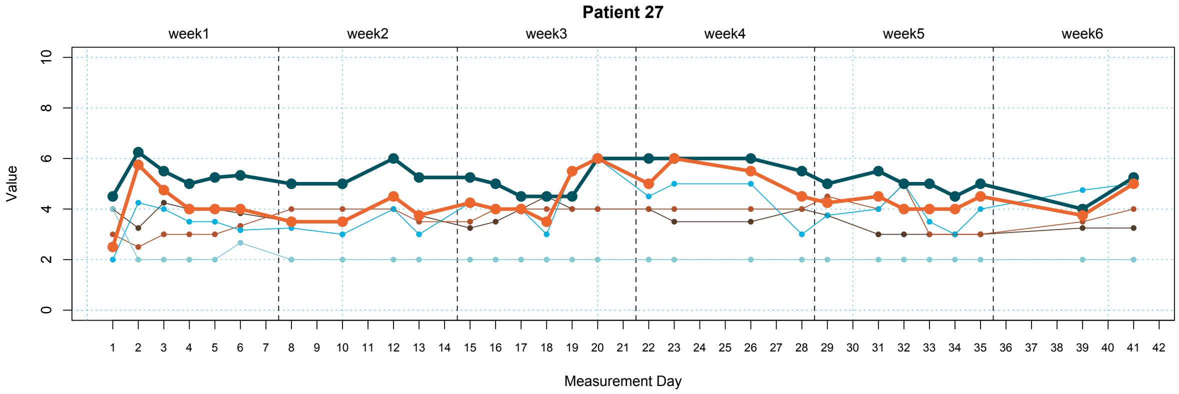
Patient 27
week1
week2
week3
week4
week5
week6
Value
Measurement Day

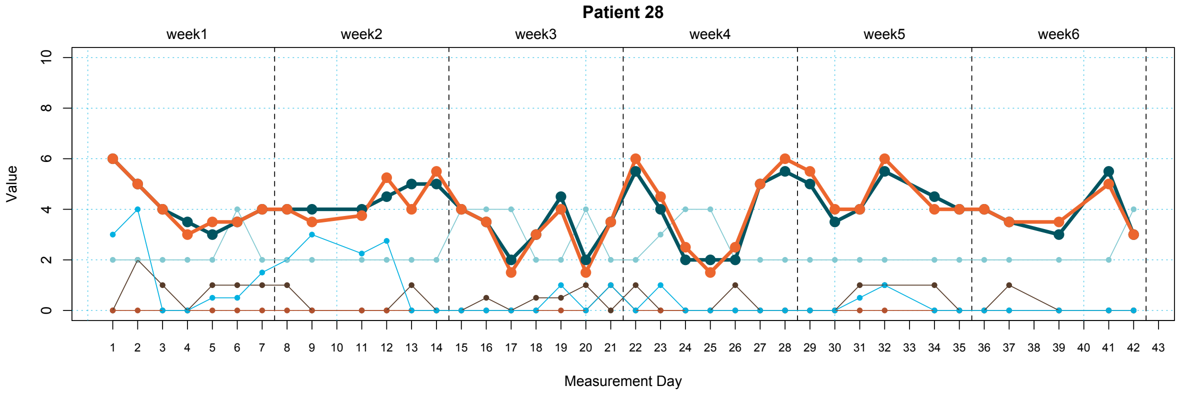
Patient 28
week1
week2
week3
week4
week5
week6
Value
Measurement Day

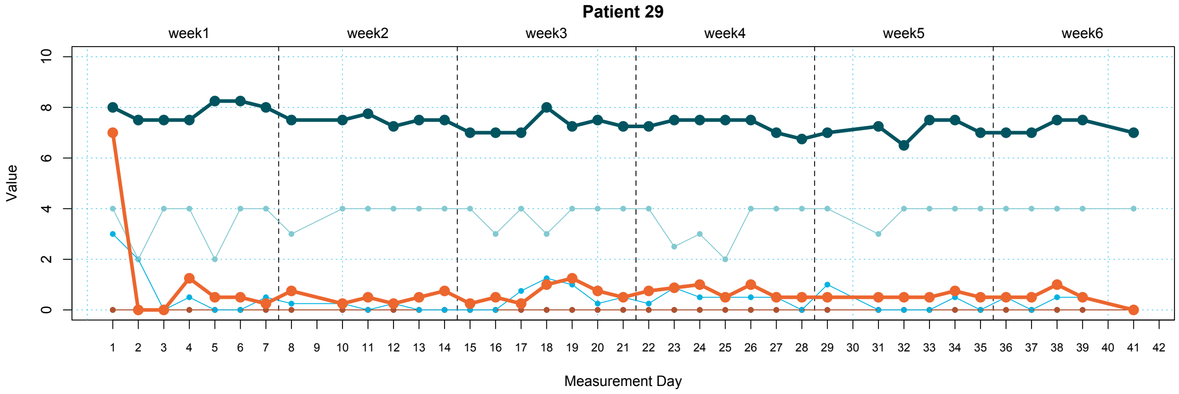
Patient 29
week1
week2
week3
week4
week5
week6
Value
Measurement Day

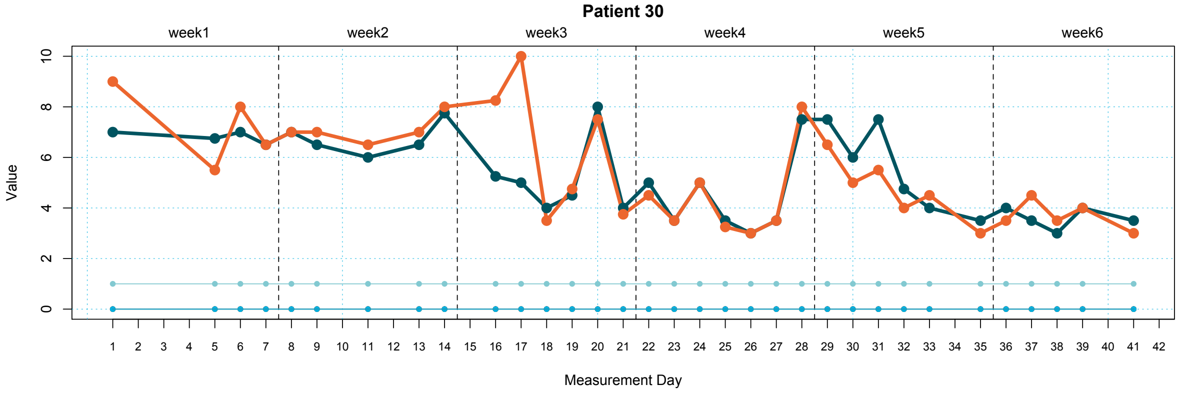
Patient 30
week1
week2
week3
week4
week5
week6
Value
Measurement Day

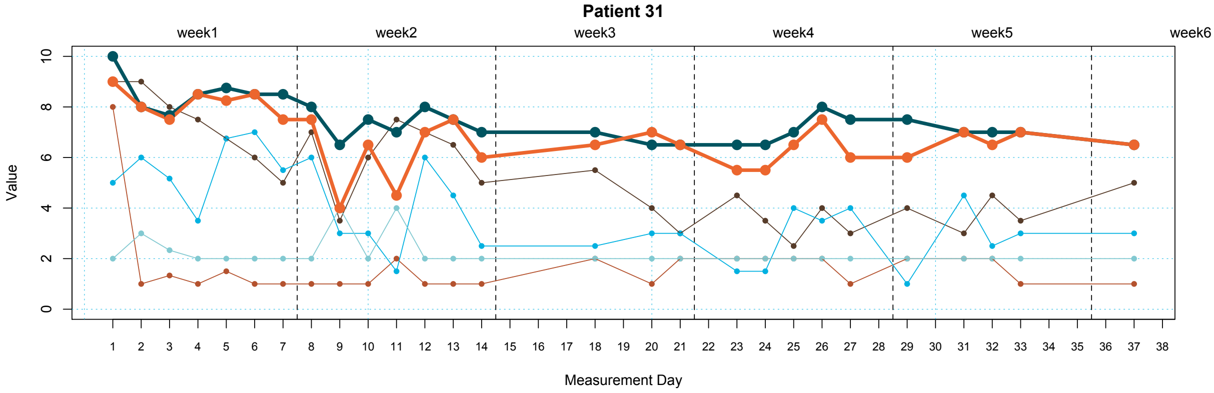
Patient 31
week1
week2
week3
week4
week5
week6
Value
Measurement Day

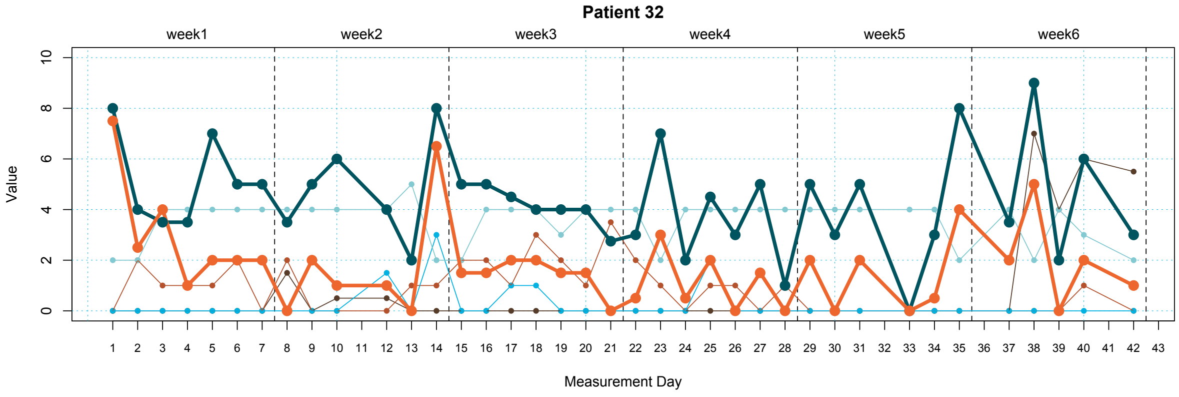
Patient 32
week1
week2
week3
week4
week5
week6
Value
0 2 4 6 8 10
1 2 3 4 5 6 7 8 9 10 11 12 13 14 15 16 17 18 19 20 21 22 23 24 25 26 27 28 29 30 31 32 33 34 35 36 37 38 39 40 41 42 43
Measurement Day

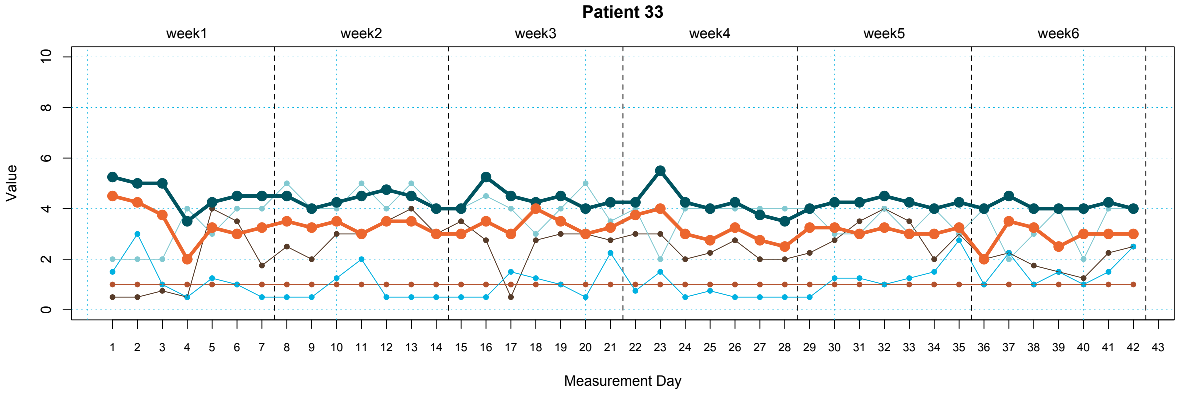
Patient 33
week1
week2
week3
week4
week5
week6
Value
0 2 4 6 8 10
1 2 3 4 5 6 7 8 9 10 11 12 13 14 15 16 17 18 19 20 21 22 23 24 25 26 27 28 29 30 31 32 33 34 35 36 37 38 39 40 41 42 43
Measurement Day

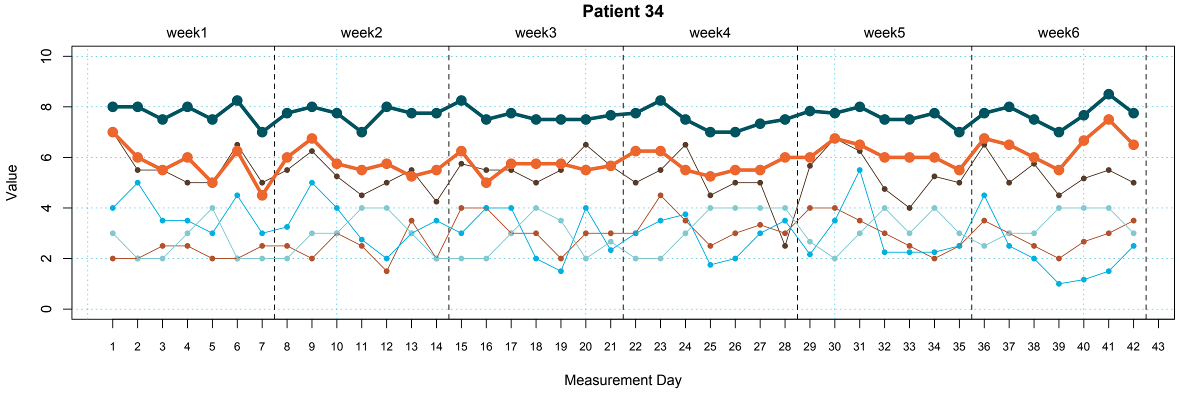
Patient 34
week1
week2
week3
week4
week5
week6
Value
0 2 4 6 8 10
1 2 3 4 5 6 7 8 9 10 11 12 13 14 15 16 17 18 19 20 21 22 23 24 25 26 27 28 29 30 31 32 33 34 35 36 37 38 39 40 41 42 43
Measurement Day

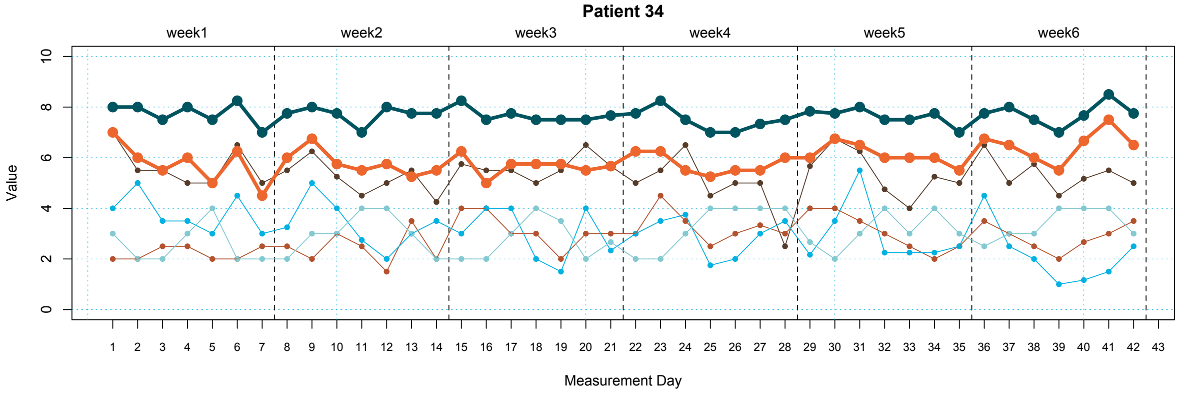
Patient 34
week1
week2
week3
week4
week5
week6
Value
0 2 4 6 8 10
1 2 3 4 5 6 7 8 9 10 11 12 13 14 15 16 17 18 19 20 21 22 23 24 25 26 27 28 29 30 31 32 33 34 35 36 37 38 39 40 41 42 43
Measurement Day

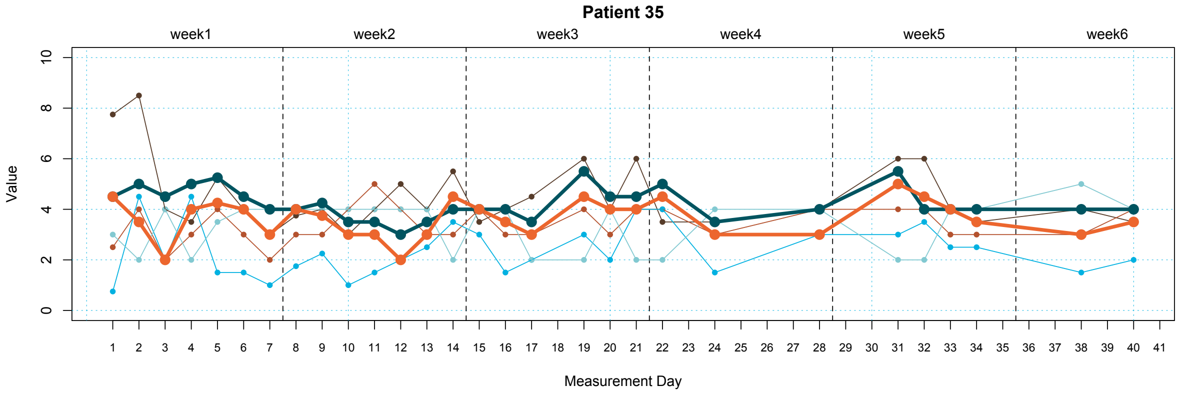
Patient 35
week1
week2
week3
week4
week5
week6
Value
0 2 4 6 8 10
1 2 3 4 5 6 7 8 9 10 11 12 13 14 15 16 17 18 19 20 21 22 23 24 25 26 27 28 29 30 31 32 33 34 35 36 37 38 39 40 41
Measurement Day

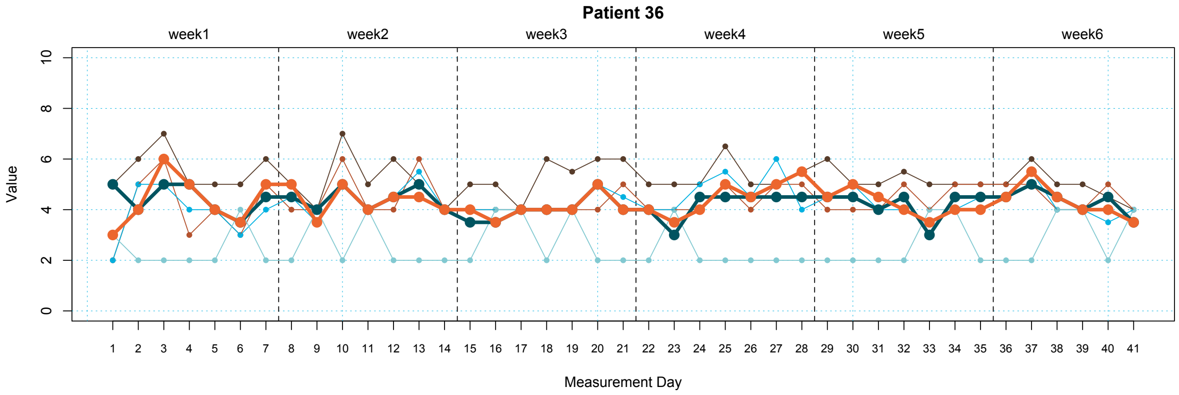
Patient 36
week1
week2
week3
week4
week5
week6
Value
0 2 4 6 8 10
1 2 3 4 5 6 7 8 9 10 11 12 13 14 15 16 17 18 19 20 21 22 23 24 25 26 27 28 29 30 31 32 33 34 35 36 37 38 39 40 41
Measurement Day

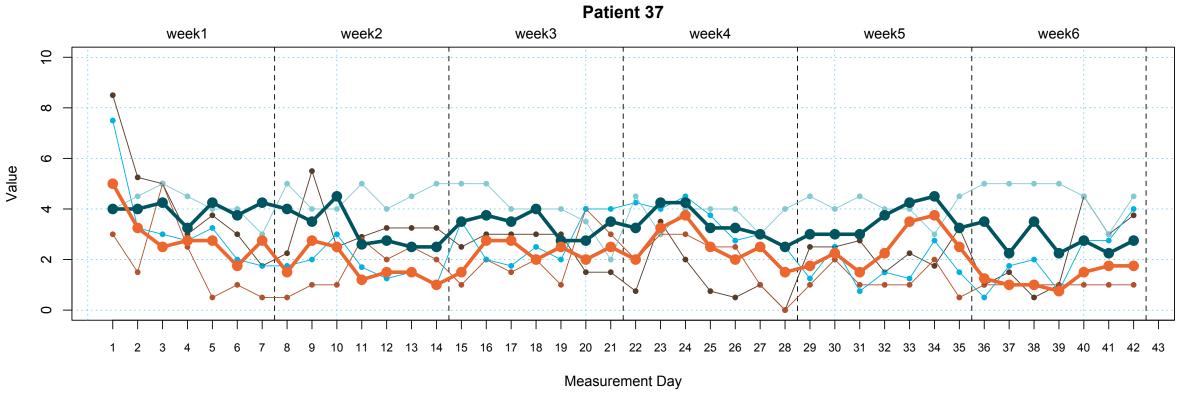
Patient 37
week1
week2
week3
week4
week5
week6
Value
0 2 4 6 8 10
1 2 3 4 5 6 7 8 9 10 11 12 13 14 15 16 17 18 19 20 21 22 23 24 25 26 27 28 29 30 31 32 33 34 35 36 37 38 39 40 41 42 43
Measurement Day

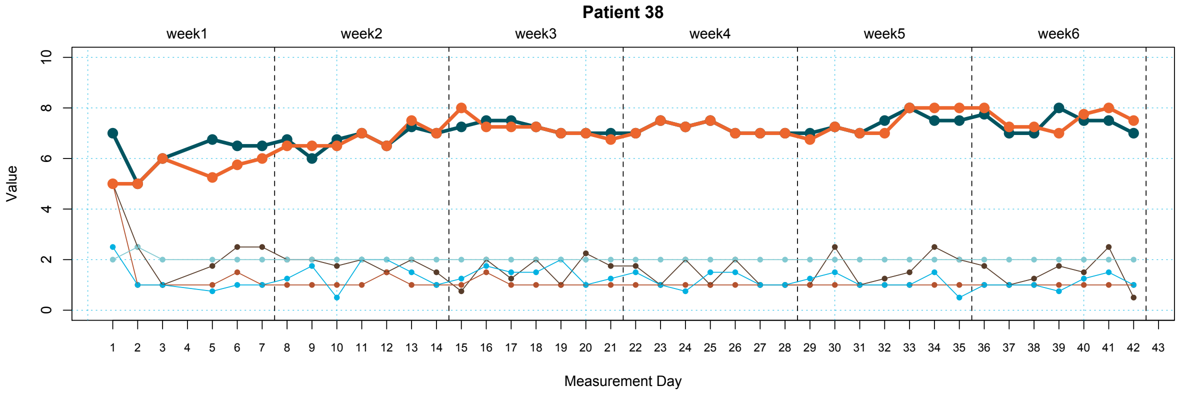
Patient 38
week1
week2
week3
week4
week5
week6
Value
0 2 4 6 8 10
1 2 3 4 5 6 7 8 9 10 11 12 13 14 15 16 17 18 19 20 21 22 23 24 25 26 27 28 29 30 31 32 33 34 35 36 37 38 39 40 41 42 43
Measurement Day

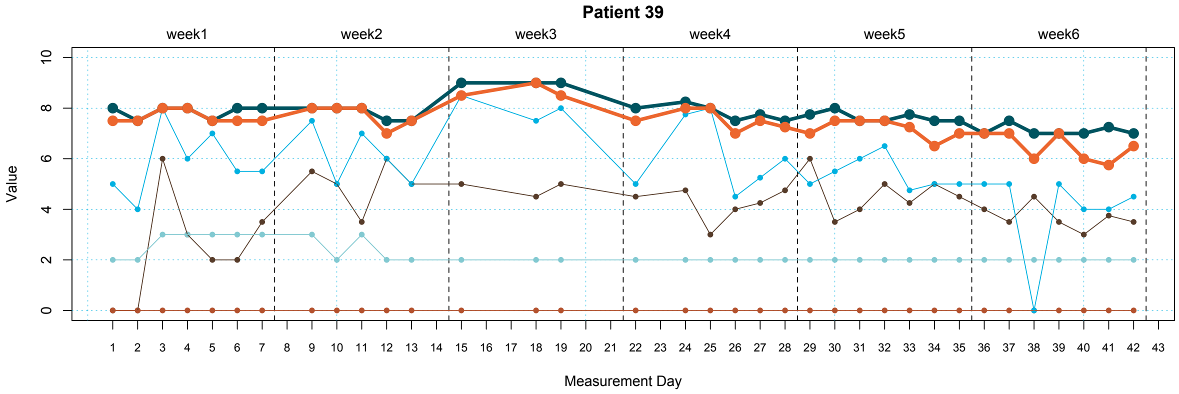
Patient 39
week1
week2
week3
week4
week5
week6
Value
0
2
4
6
8
10
1 2 3 4 5 6 7 8 9 10 11 12 13 14 15 16 17 18 19 20 21 22 23 24 25 26 27 28 29 30 31 32 33 34 35 36 37 38 39 40 41 42 43
Measurement Day

S2. Supplemental Material. Overview of the linear regression estimated in the study group. Estimates are summerized and mean ± standard deviation are provided in the table together with the range of estimates [min; max] .

Symptom	Intercept	Slope
Tinnitus Loudness	5.72 ± 1.79<br>[3.16; 9.13]	-.023 ± .027<br>[-.098; .030]
Tinnitus Distress	4.70 ± 2.13<br>[.97; 8.84]	-.018 ± .036<br>[-.118; .060]
Stress	2.65 ± 1.89<br>[.07; 7.84]	-.022 ± .033<br>[-.107; .045]
Tension in the jaw muscles	2.17 ± 1.82<br>[.022; 7.81]	-.008 ± .029<br>[-.087; .051]
Tension in the neck muscles	3.00 ± 2.29<br>[-1.06; 8.16]	-.013 ± .037<br>[-.144; .085]
Mood	2.84 ± .85<br>[1.00; 4.44]	.000 ± .016<br>[-.055; .035]
